# The Spatial Ecology of Nuisance Crocodiles: Movement Patterns of Relocated American Crocodiles (*Crocodylus acutus*) in Guanacaste, Costa Rica

**DOI:** 10.3390/ani14020339

**Published:** 2024-01-22

**Authors:** Tyler Steven Coleman, Wray Gabel, Michael Easter, Maggie McGreal, Mahmood Sasa Marin, Davinia Beneyto Garrigos, Christopher M. Murray

**Affiliations:** 1 Department of Biological Sciences, Southeastern Louisiana University, Hammond, LA 70401, USAmaggie.mcgreal@selu.edu (M.M.); 2 Scales and Tails of Ohio, Lakewood, OH 44107, USA; 3 Instituto Clodomiro Picado, Facultad de Microbiología, Universidad de Costa Rica, San Jose 11403, Costa Rica; msasamarin@gmail.com; 4 Centro Investigaciones en Biodiversidad y Biologia Tropical, Universidad de Costa Rica, San Jose 11501, Costa Rica; 5 Escuela de Biología, Universidad de Costa Rica, San Jose 11501, Costa Rica; davinia.beneyto@gmail.com

**Keywords:** spatial ecology, American crocodile, human–wildlife conflict, relocation

## Abstract

**Simple Summary:**

We aimed to evaluate the efficacy of nuisance crocodile relocations using passive spatial quantification in Guanacaste, Costs Rica. Nuisance relocated crocodiles and wild crocodiles were fit with Iridium satellite trackers and movements of all individuals were monitored over months. Nuisance relocated crocodiles either returned to the area of nuisance, or potentially attempted to return, in short time frames. These results highlight the need for alternative management strategies.

**Abstract:**

Anthropogenic alterations of the environment have increased, highlighting the need for human–wildlife coexistence and conflict mitigation. Spatial ecology, and the use of passive satellite movement technology in particular, has been used to identify patterns in human–wildlife conflict as a function of shared resources that present potential for dangerous situations. Here, we aim to remotely identify patterns indicative of human–crocodile conflict in Guanacaste, Costa Rica by exploring site fidelity and diverse modes of movement (i.e., land and water) across space between nuisance (relocated) and non-nuisance (wild) crocodiles. Advanced satellite remote sensing technology provided near-constant movement data on individuals at the regional scale. Telonics Iridium SeaTrkr-4370-4 transmitters were used with modified crocodilian fitting. Results indicate that relocated crocodiles exhibited large-scale movements relative to wild crocodiles. Nuisance relocated crocodiles either returned to the area of nuisance or potentially attempted to in short time frames. The results presented here highlight the need for alternative management strategies that facilitate relocation efficacy.

## 1. Introduction

Quantifying the spatial ecology of vertebrates has elucidated patterns pertinent to life history, niche breadth, and population persistence. Emerging technologies have allowed passive data acquisition, permitting remote capabilities that acquire data on animal spatial use patterns unbiased by human approach and observation. Such data have improved natural science hypothesis testing pertaining to resource use and reproduction [1], as well as conservation assessment in the context of anthropogenic land use [2] from an applied science perspective. Anthropogenic influences on the biophysical environment have increased dramatically over the past few decades [3], highlighting the critical importance of facilitating human–wildlife coexistence for conservation [4,5,6].

Spatial ecology in the applied sciences has focused primarily on two fronts: human–wildlife conflict and conservation reintroductions. Human–wildlife conflict approaches track space use associated with shared resources, such as water [7] and crops [8,9] that present potential for dangerous situations or crop destruction based on animal movement patterns. This approach has been especially useful in identifying human activities and landscape alterations that facilitate large carnivore movements into human populated areas [7] and understanding how animals modify their behavior and activity patterns to exploit resources, in order to mediate coexistence outside protected lands [8,9]. Conservation-based repatriation has also benefited from remote spatial sensing, assessing navigation, and use of new environments [6,10], as well as the importance of social cues associated with movement in reintroduction protocols [11]. Little work thus far, however, has used remote sensing to evaluate relocation protocols in the context of human–wildlife conflict.

The Pacific versant of Costa Rica harbors one of the most unique and diverse aquatic landscapes in the world, supporting marine coastline, estuarine habitat, seasonally ephemeral wetlands, freshwater rivers, and a network of irrigation canals. Equally complex is the ecology and physiology of, and the human impact on, the region’s apex predator, the American crocodile (*Crocodylus acutus*). Aquaculture facilities have served as an anthropogenic food source for crocodiles—an introduction source of non-native tilapia (*Oreochromis niloticus* and *O. mossambicus* [12])—and an interface for human–crocodile conflict within the facilities themselves. Recent work suggests that continuous tilapia introduction serves as a pollutant source for methyltestosterone (MT), a synthetic androgen used in aquaculture practices to increase growth rates and overall sizes of farmed fish [13]. Demographic research has identified a unique male-biased sex ratio in local crocodile populations that stems from MT exposure [13,14,15], resulting in hermaphroditic individuals [16]. Of additional concern is the influence of aquaculture facilities on crocodile movement, potentially endogenously biased by MT exposure, but further, the anthropogenic recruitment of crocodiles to aquaculture facilities for food, causing subsequent human–crocodile conflict.

Relocation efforts from aquaculture facilities to address this issue include the maintenance of a capture and relocation team that isolates and relocates nuisance crocodiles to Palo Verde National Park [17]. Crocodiles, however, frequently return to the nuisance area, posing a danger to workers and surrounding humans [17]. Additionally, human conflict with crocodiles in the Tempisque Basin ecosystem has increased as the distributions of humans, aquaculture facilities, and wildlife increasingly overlap, and crocodile attacks continue to become more frequent. As such, mediating this conflict requires the identification of dispersal routes that lead to close proximity between humans and crocodiles. Specifically, the movement patterns and return routes of relocated crocodiles compared to the movement patterns of wild crocodiles are a key component to conflict mitigation and efficacy of the relocation program.

Here, we aim to remotely identify patterns indicative of human–crocodile conflict by I. exploring site fidelity and diverse modes of movement across space between nuisance (translocated) and non-nuisance (resident) crocodiles, and II. identifying aquatic infrastructure routes that lead to human–crocodile conflict in aquaculture facilities to evaluate solution-based containment. Specifically, we aim to determine variation in site fidelity and modes of movement across space between relocated and wild crocodile populations. Identifying these annual movement patterns among cohorts is the first step in understanding how natural history in space and time interacts with anthropogenic disturbance and may result in conflict. We hypothesize that relocated animals disperse the farthest from their release site in protected areas. Lastly, we aim to identify dispersal routes that lead to human–crocodile conflict in and around aquaculture facilities to better manage this growing tension. The proximity of large–scale aquaculture facilities to protected natural areas has led to high rates of dangerous biotic exchange; crocodiles access aquaculture ponds so readily that the expensive nuisance quarantine and relocation protocol is warranted. We hypothesize that translocated crocodiles efficiently gain access to the facilities using specific natural and artificial waterways. Ultimately, we aim to elucidate patterns useful for effective conservation protocols that benefit these aquatic systems and human–crocodile conflict.

## 2. Materials and Methods

**Figure 1:**
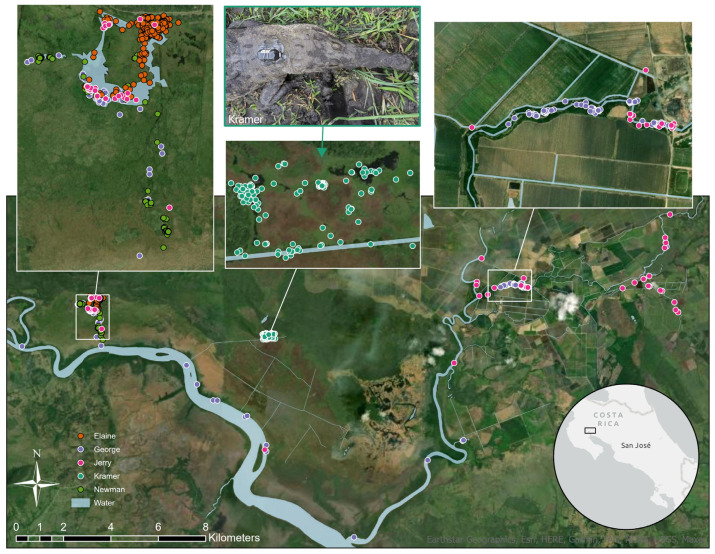
Tempisque River basin in Costa Rica with Palo Verde National Park depicted between the two rivers. Each crocodile is represented by a unique point color. The top-left, top-right, and middle windows show zoomed-in portions of the basin for visual aid. The top-middle window displays a crocodile (Kramer) with an attached satellite tag. The top-left insert also depicts the release location for all translocated crocodiles.

Five American crocodiles (four male and one female between 261 cm and 305 cm total length; Table 1) were captured using the harmless snare pole methodology described in Murray et al. [13]. Three males (George, Jerry, and Newman) were captured from aquaculture ponds, relocated, and released at Palo Verde National Park on 14 May 2021 at the onset of the rainy season (mid-May to November). These three males make up the translocated treatment group. Additionally, one wild male (Kramer) and one wild female (Elaine) were captured in Palo Verde National Park’s ephemeral wetlands on 11 May 2021 and 12 May 2021, respectively (Table 1). All animals were fitted with the remote sensing technology described below and released at Palo Verde National Park the same day (Figure 1). Wild-caught animals were released within one hour of capture at the capture site. Animals captured in the aquaculture ponds were relocated by truck and released the same day.

Advanced satellite remote sensing technology was used to efficiently address both objectives simultaneously. The satellite platform terminal transmitter data collection system was used to provide near-constant movements of individuals at the regional scale. Telonics Iridium SeaTrkr-4370-4 transmitters were used with modified crocodilian fitting. Sensors were programmed to attempt signal transmission for 10-min durations at 0800 h and 2300 h UTC every day for one calendar year. Captured animals were restrained while morphometrics and blood samples were taken using whole blood (3cc) using 1.5″ 21-gauge needles and stored in heparinized vacutainers. The nuchal rosette was disinfected with betadine and a 0.5 mL injection of ketoprofen solution (2 mg/kg) was administered in the front limb. A local Lidocaine anesthetic (2% Lidocaine HCL 20 mg/mL, 7–8 mL) was administered subcutaneously to the nuchal rosette area (the four nuchal scutes and the area between them). Transmitters were secured to the nuchal scutes with wires running through the scutes using a marine grade DevCon epoxy mold and 1.65-mm wires crimped with lead sleeves (Figure 1). All equipment was disinfected in a 95% isopropyl alcohol bath before application. This protocol is modified from [18]. After attachment, animals were allowed 30 min of restrained monitoring and more than one hour to recover without restraint but all made their way to the water within one hour.

All statistical analyses were conducted in R [19], and an alpha value of 0.05 was used to determine statistical significance. Site fidelity here is referred to as an individual’s tendency to return to a previously visited location. We use the term “home range” here to represent the area traversed by an individual in its normal activities, as ecological theory has long stressed the connected principles of space usage, habitat selection, resource selection, and home range [20]. Data received from ARGOS for each American crocodile was overlaid onto the Tempisque River basin shapefile after being transformed to the World Geodetic System of 1984 ellipsoid, Universal Transverse Mercator Zone 16. Brownian bridge movement models [21] were used to assess individual site fidelity, home range, and movement patterns using the adehabitatHR package [22]. A different perspective on home range analysis is offered by Brownian bridge models, which explicitly include movement trajectories in the interpretation of the home range, predicated on the notion of a conditional random walk process between successive locations. This assigns a mechanistic role to observed movement in home range estimation, unlike in kernel methods [23]. Most importantly, these models do not assume locations are independent (unlike kernel methods), resulting in estimated connected home ranges [23], which is imperative when assessing individual space-use and movement patterns. Time is available for each GPS location, which allowed the use of a type II trajectory when estimating step lengths (i.e., distance moved between time *t* − 1 and *t*) and turning angles along the random walk process between successive locations of each individual. The adehabitatLT package [24] was used to convert the data to trajectory estimates. A path selection alternative trajectory was not necessary, as the dataset and analysis did not contain temporal autocorrelation, as seemingly one main path (i.e., the river) was used for individual movement over the short data collection time period, if moving a significant distance at all. Sigma one for the Brownian bridge was estimated following recommendations [23], and sigma two was quantified by averaging the horizontal error in the location data for each individual in the ARGOS dataset. The 50% and 95% home range utilization distributions were estimated for the relocated individuals, while the 50% and 75% distributions were estimated for the wild individuals due to the small spatial extent of their detected movements. An analysis of variance (ANOVA [25]) and Tukey’s honest significant difference (HSD [26]) test were used to assess if there were differences in the distances traveled between detections among the five American crocodiles.

## 3. Results

The total distance traveled for relocated crocodiles was 6991.35 (Newman), 70,448.88 (Jerry), and 89,248.92 m (m; George), and the average estimated distance traveled between time steps was 42.12 (Newman), 667.04 (George), and 880.61 m (Jerry) according to the type II random walk trajectory. The total distance traveled for wild crocodiles (Kramer and Elaine) was between 6601.49 and 34,245.47 m, and the average estimated distance traveled between time steps ranged from 34.03 to 77.48 m, respectively. The total length (TL) of the individuals ranged from 258 cm (Kramer) to 305 cm (Newman), and snout-vent length (SVL) ranged from 138 cm (Kramer) to 158 cm (Newman; Table 1).

Crocodiles George and Jerry both traveled significantly further after relocation than the wild crocodiles Kramer (George: F_1,4_ = 32.89, 632.01 m ± 242.74 m [95% CI], *p* < 0.001; Jerry: F_1,4_ = 32.89, 846.58 m ± 1406.03 m, *p* < 0.001) and Elaine (George: F_1,4_ = 32.89, 588.56 m ± 213.11 m, *p* < 0.001; Jerry: F_1,4_ = 32.89, 803.13 m ± 262.56 m, *p* < 0.001; Table 2). There was no significant difference in the distance traveled between the wild crocodiles Kramer and Elaine (F_1,4_ = 32.89, 43.45 m ± 186.11 m, *p* = 0.97) or between the translocated crocodiles George and Jerry (F_1,4_ = 32.89, 214.57 m ± 305.33 m, *p* = 0.31; Table 2 and Table 3). However, the behavior of the translocated crocodile Newman more closely resembled that of the wild crocodiles, in that his total distance traveled was not significantly different from that of Kramer (F_1,4_ = 32.89, 8.09 m ± 228.48 m, *p* = 0.99) or Elaine (F_1,4_ = 32.89, 35.36 m ± 125.99 m, *p* = 0.99), and was significantly less than George (F_1,4_ = 32.89, 623.92 m ± 250.96 m, *p* < 0.001) and Jerry (F_1,4_ = 32.89, 838.49 m ± 294.11 m, *p* < 0.001; Table 2 and Table 3). The only female crocodile (Elaine), which was wild, did not differ in distance traveled from the other wild crocodile, Kramer (F_1,4_ = 32.89, 43.45 m ± 186.11 m, *p* = 0.97) despite an apparent higher estimated total distance and average distance traveled.

**Figure 2:**
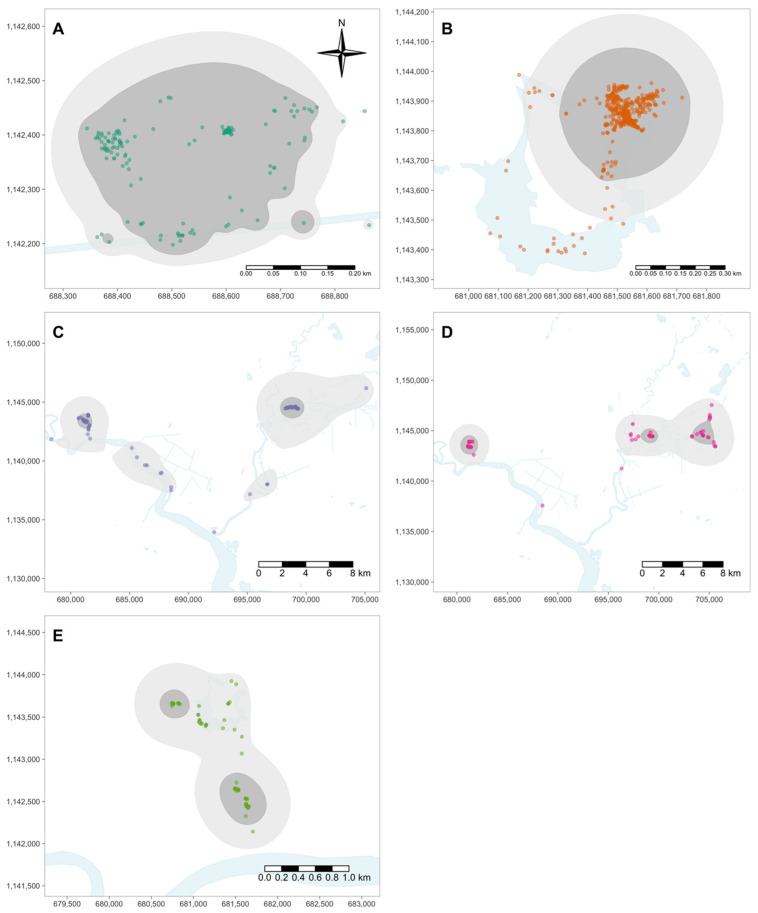
Home range predicted from the Brownian bridge movement models for each individual: Kramer (resident; (**A**)), Elaine (resident; (**B**)), George (translocated; (**C**)), Jerry (translocated; (**D**)), Newman (translocated; (**E**)). Points represent the successfully received GPS data. Shaded areas are the 50% (dark gray), 75% ((**A**,**B**); light gray), and 95% ((**C**–**E**); light gray) estimated habitat utilization distributions.

**Figure 3:**
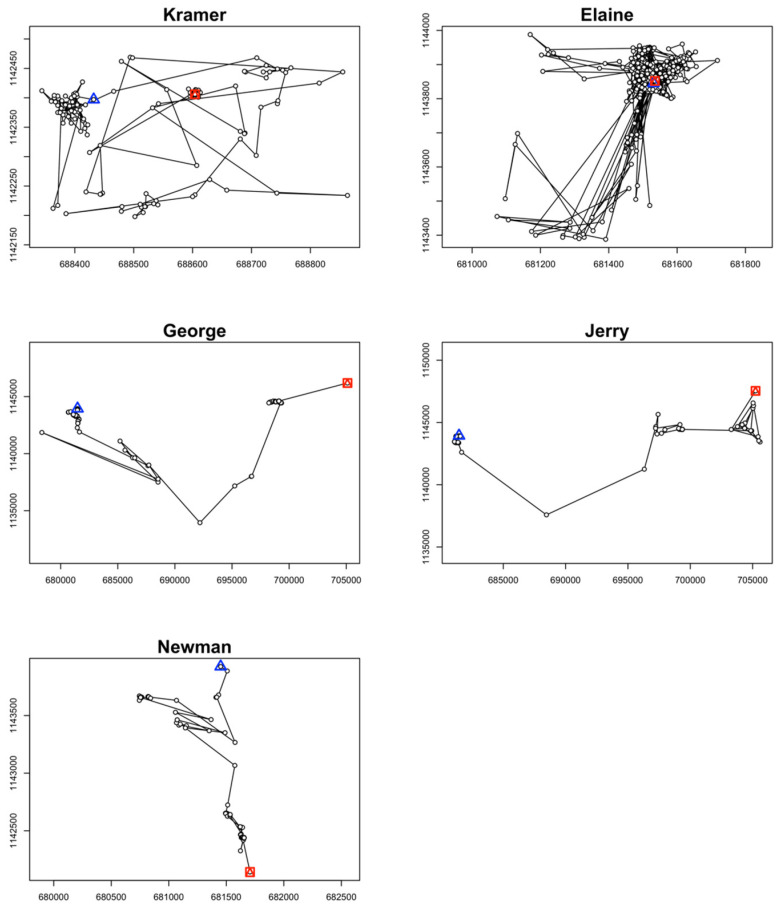
Initial (blue triangle) and final (red square) locations of each individual based on the type II random walk trajectory used for the Brownian bridge movement models that were used to estimate home range (Figure 2).

Home range size for Kramer was 0.11 km^2^ for the 50% habitat utilization and 0.18 km^2^ for the 75% habitat utilization distributions (Figure 2). Similarly, for the other wild caught crocodile, Elaine, home range size was 0.15 km^2^ for the 50% and 0.36 km^2^ for the 75% habitat utilization distributions (Figure 2). For both crocodiles, habitat utilization was concentrated into a single large cell or had limited supplementary cells (Figure 2), which is also evident in the translocation trajectory depicted in Figure 3 (top two panels), where both individuals moved short distances within their home range. Kramer and Elaine moved a net 6601.49 m and 34,245.47 m, respectively, throughout the duration of the study (Figure 3; Table 2). The relocated crocodile Newman had a home range of 0.34 km^2^ for the 50% and 2.26 km^2^ for the 95% estimated habitat utilization distributions, where observations were concentrated into two distinct foci. Newman appeared to be moving towards the Tempisque River like the other relocated crocodiles before successfully received data ceased. The home ranges of the other two relocated crocodiles, George and Jerry, were estimated to be 3.94 and 8.02 km^2^ and for the 50% habitat utilization and 76.57 and 64.31 km^2^ for the 95% habitat utilization distributions, respectively (Figure 2). George and Jerry traveled 89,248.92 and 70,448.88 m throughout the duration of the estimated trajectory (Figure 3; Table 2).

## 4. Discussion

The average distance and total distance traveled varied between translocated and wild (resident) crocodiles. Relocated animals either traveled back to the original point of capture (~63 river km) or potentially attempted to. This attempt, by Newman, followed the same path as the other two relocated crocodiles by leaving the humedal wetland to the south, entered the Tempisque River, and tracking was lost. All translocated animals made the same initial movement after release. Wild crocodiles tracked in Palo Verde National Park remained close to their point of capture and constrained movements to relatively small home ranges. Relocated individuals showed no signs of home range movements and all immediately attempted to return to their original point of capture. Data presented here suggest that relocation efforts are futile solutions to nuisance crocodile problems, at least at the distance presented herein, and that crocodiles retain remarkable ability to navigate long distances with precision. Potential sensory cues that aid in navigation, such as olfactory stimuli or individual movement history, are not clear and should be further investigated in this species.

Homing behavior after translocation is not unique to this study. Read et al. [27] reported rapid return to capture sites in translocated saltwater crocodiles (*Crocodylus porosus*) in coastal Australia, over distances up to 411 km. Fukuda et al. [28] reported similar trends in the same species, complicating management practices and protocols, however, they reported that peninsular landforms impeded effective homing ability, a challenge that appears non-existent for the American crocodiles examined in the present study. Further, American crocodiles have exhibited variation in movements among seasons, cohorts, and sex [29]—patterns that were ignored by nuisance relocated animals here (readily traveled across land) and not tracked in wild, resident crocodiles. Campbell et al. [30] also investigated potential human–crocodile interaction in space and found that previously tagged saltwater crocodiles from various habitats frequented human–occupied habitat.

Specific to *Crocodylus acutus* translocations, Beauchamp et al. [31] and Brunell et al. [32] investigated the homing capabilities of nuisance crocodiles moved away from their capture site by varying distances. Brunell et al. [32] used the South Florida sink population to evaluate return capability to capture site, associating habitat type to movement patterns in seven translocated individuals compared to movements of control resident individuals. Like the present study, ref. [32] also used home range estimations to compare movement patterns in translocated to resident animals. Interestingly, four of seven translocated crocodiles did not return to within 2 km of their original capture site, although return motive was assigned to one additional crocodile. All returning animals were translocated <45 km from original capture site, a distance about 20 km shorter than the crocodiles in the present study. Brunell et al. [32] is less comparable to the present study than expected given that the vast majority of their study area is protected wetlands, however the highest density of crocodiles persists in anthropogenic cooling canals near urban areas. This is evidenced by the negative regression coefficients for state transition probabilities when crocodiles inhabited ‘anthropogenic wet’ and ‘upland forested’ habitats [32] (p. 15). Our data suggest that animals in Guanacaste rapidly return to their original capture site across farther distances over roughly the same time period, predominantly using natural waterways and ephemeral marshes. 

From a management perspective, data presented here warrant reconsideration of protocols for nuisance crocodiles in Guanacaste, Costa Rica. If relocation is to become an effective practice, nuisance animals require much more distant relocation or relocation to a place that presents an impenetrable barrier between release and capture site. Relocation to an educational facility, however, may mitigate the shortfalls of current practices and be economically efficient. Future directions in the Tempisque Basin should expand sampling to effectively assess variation in spatial ecology between sexes, among varying environmental androgen exposure, seasonality, and translocation distance. Future spatial analyses should include the use of calibrated tri-axial accelerometers to pair behaviors with individual locations. Additionally, the common use of the fangueo wetland restoration technique in this region remains unevaluated in the context of crocodile resource use, population dynamics, and movement ecology. This management technique uses specialized tractors to bury invasive cattail (*Typha* sp.), which clears the wetland for native flora and fauna [33]. Crocodiles may be disturbed by this technique and avoid managed areas or cause them to preferentially select managed habitat. A better understanding of these complex factors influencing crocodile movement may provide insight useful to refine human–crocodile conflict management protocols.

## 5. Conclusions

Human–wildlife conflicts are increasing in prevalence worldwide, and ecological dynamics directly relate to the loss of human lives [34]. Specific to some Costa Rican locations, crocodiles can occur in dangerous proximity to towns and businesses, and management updates are necessary [34]. The movement and habitat range (i.e., land, water) reported herein indicate significantly amplified movement patterns of relocated American crocodiles, consistently putting these animals in positions to have a higher probability of human interaction (farms, irrigation canals, fishing, and swimming areas). The relocation process is mainly necessary due to the anthropogenic alteration of the environment through the agriculture and aquaculture waterways, which are together increasing the prey availability of the American crocodile near local human populations [14,15]. Management and decision-making concerning crocodilian movement patterns (i.e., human–wildlife conflict), agricultural land use, and release of xenobiotics into the ecosystem are essential, while maintaining livelihoods in this region and across the globe.

It is important for us to understand the relationship between economic choices and potential impacts on human–nature interactions if we wish to limit the impacts of anthropogenic influences on crocodilian populations and expand the conservation of these animals. Therefore, we urge future research on crocodilian conservation and management to treat these complex problems as coupled human and natural systems [3]. Despite growth in global crocodilian research focusing on connections between the ecological e.g., [13,35,36], social e.g., [37,38,39,40], and economic e.g., [41,42,43,44] factors influencing and influenced by, crocodilians, there are still knowledge gaps surrounding these linkages, including their magnitude and extent, underlying causes, and ultimate effects from local to global scales. Integration of (1) patterns and processes that link humans and natural systems, (2) reciprocal interactions and feedbacks between humans and the environment, and (3) understanding scalar-interactions between human and natural components (e.g., large-scale phenomena that emerge from the interactions of multiple agents at the local scale, influencing local systems [45]) is required to address the increased complexity and help prevent the negative consequences that may occur due to fundamentally new and rapid changes [3,46,47] on successful crocodilian conservation.

## Figures and Tables

**Table 1:** Characteristics of American crocodiles tagged with Telonics Iridium SeaTrkr-4370-4 transmitters. Abbreviations include the transmitter identification number (ID) that correlates with the date each individual was tagged in 2021 (Date), each crocodile’s name (Name) in the dataset, if the crocodile was translocated or caught in the wild (resident; Treatment), total length (TL; cm) and snout-vent length (SVL; cm) of the individual.

Date	ID	Name	Treatment	SVL (cm)	TL (cm)	Sex
11 May 2021	716811A	Kramer	resident	138	258	Male
12 May 2021	716807A	Elaine	resident	140	261	Female
14 May 2021	716739A	George	translocated	147	291	Male
14 May 2021	716812A	Jerry	translocated	155	293	Male
14 May 2021	716799A	Newman	translocated	158	305	Male

**Table 2:** Spatial data of American crocodiles tagged with Telonics Iridium SeaTrkr-4370-4 transmitters. Abbreviations include the transmitter identification number that correlates with each crocodile’s name in the dataset (ID; Name), if the crocodile was translocated or caught in the wild (resident; Treatment), the total number of successfully received observations (*N* obs), the number of months data were collected (# Months), the estimated total distance travelled from the type II random walk trajectory (Tot Dist; m), and the average estimated distance travelled between time steps from the trajectory (Avg Dist; m).

ID	Name	Treatment	*N* obs	# Months	Tot Dist (m)	Avg Dist (m)
716811A	Kramer	resident	195	11	6601.49	34.03
716807A	Elaine	resident	443	9	34,245.47	77.48
716739A	George	translocated	135	4	89,248.92	667.04
716812A	Jerry	translocated	81	4	70,448.88	880.61
716799A	Newman	translocated	167	4	6991.35	42.12

**Table 3:** Tukey’s honestly significant difference testing of the analysis of variance results examining the statistical differences in distance traveled (m) among the five individuals. Bolded groups signify statistically significant differences (*p* < 0.05), with the estimated upper and lower 95% confidence intervals around the mean difference (m). A negative value indicates the first group travels an average distance of [Mean Diff; 2.5–97.5%; 95% CI] m less than the second group per time step. Individuals with superscript ^t^ are translocated.

Compared Crocodiles	2.5% CI	Mean Diff	97.5% CI	*p*-Value
**George** ^t^ **–Elaine**	375.45	588.56	801.67	<0.001
**Jerry** ^t^**–Elaine**	540.57	803.13	1065.70	<0.001
Kramer–Elaine	−229.56	−43.45	142.66	0.97
Newman ^t^–Elaine	−232.078	−35.36	161.35	0.99
Jerry ^t^–George ^t^	−90.75	214.57	519.90	0.31
**Kramer–George** ^t^	−874.75	−632.0084	−389.27	<0.001
**Newman** ^t^**–George** ^t^	−874.88	−623.92	−372.96	<0.001
**Kramer–Jerry** ^t^	−1133.73	−846.58	−559.45	<0.001
**Newman** ^t^**–Jerry** ^t^	−1132.61	−838.49	−544.38	<0.001
Newman ^t^–Kramer	−220.39	8.088	236.57	0.99

## Data Availability

The data presented in this study are available on request from the corresponding author. The data are not publicly available due to proprietary locations.
